# Effects of Group-Play Moderate to Vigorous Intensity Physical Activity Intervention on Executive Function and Motor Skills in 4- to 5-Year-Old Preschoolers: A Pilot Cluster Randomized Controlled Trial

**DOI:** 10.3389/fpsyg.2022.847785

**Published:** 2022-06-15

**Authors:** Jing Bai, Heqing Huang, Huahong Ouyang

**Affiliations:** ^1^College of Preschool Education, Capital Normal University, Beijing, China; ^2^College of Teacher Education, Capital Normal University, Beijing, China

**Keywords:** executive function, group play, physical activity, preschool education, motor skills

## Abstract

The aim of the present study is to examine the effect of group-play intervention on executive function (EF) in preschoolers. This group-play intervention was integrated as moderate to vigorous physical activity and cognitively loaded exercise to promote EF in preschoolers. An 8-week group-play MVPA intervention program, consisting of a series of outdoor physical and cognitively loaded games, was designed to improve preschoolers’ EF. This intervention program was implemented in group-play form, and conducted by teachers who received standardized training before the intervention. Two classes of second grade preschoolers (*N* = 62) were randomly allocated to experimental (*n* = 30, *M*_*age*_ = 4.16, *SD* = 0.29) and control (*n* = 32, *M*_*age*_ = 4.7, *SD* = 0.43) groups. The intervention group received the intervention three times a week, while the control group exercised as usual in preschool. Before, in the middle of, and after the intervention, 10-m running, standing broad jump, throwing, body flexion, balance beam, and skip jump were assessed as tests of motor skills. In addition, three components of EF were measured separately before, in the middle of, and after the intervention: inhibitory control was assessed by using the silly sound Stroop task, working memory was tested using the empty house task, and shifting was assessed using the dimensional change card sorting task. Although both groups showed an increasing trend in terms of motor skills and EF during the intervention, the increasing amounts of the intervention group were significantly higher than the control group. The findings of the present study suggested that group-play intervention has positive effects on aspects of EF in addition to motor skills in preschoolers.

## Introduction

Nowadays, the importance of physical activity (PA) in young children’s physical and mental development is increasingly acknowledged by educators and parents. Accordingly, government policy also points to increasing PA for preschool- and school-aged students. For example, the World Health Organization (WHO) recommends that preschool-aged children engage in at least 180 min of physical activity per day (60 min of which should be moderate to vigorous intensity physical activity [MVPA]), whereas school-age children are recommended to engage in 60 min of MVPA per day (Onis, 2020). Increased levels of PA, especially MVPA, are associated with improved health, cognitive, and academic outcomes for preschool-age children (Gu, 2015; Zhang et al., 2020). Meta-analytic and narrative literature reviews have documented that MVPA is specifically associated with executive function (EF) in adults, adolescents, and school-age children and preschoolers (Palmer et al., 2013; Egger et al., 2019; Koorts et al., 2019; O’Brien et al., 2021). The benefits of increased MVPA for improved academic outcomes may be due, in part, to the impact of MVPA on improved EF (Erickson et al., 2019). However, studies involving preschool-aged children indicated most preschoolers failed to reach the recommended requirement and preschoolers spend their time mostly in a sedentary state (Lindsay et al., 2017; Stone et al., 2019; Chang and Lei, 2021). Children need help to be more active. Schools, preschools, and child care are critical venues to assist children to meet daily recommendations (Aivazidis et al., 2019; Carson et al., 2020).

Executive function refers to a subset of top-down cognitive control processes for goal-directed behavior (Friedman and Miyake, 2017). Scholars believe that the main structures of EF of preschool children are inhibitory control, working memory, and shifting (McNeill et al., 2018; Jusienė et al., 2020). Inhibitory control is the ability to inhibit dominant responses and to restrain persuasive thoughts and behaviors (Diamond, 2000). Working memory refers to a system which stores and processes information simultaneously for complex cognitive tasks (Morra et al., 2018). Shifting, based on inhibitory control and working memory, is the ability to rapidly change tasks, operations, mental sets, or strategies (Diamond, 2000; Dan et al., 2021).

As an essential factor of learning and development, EF is fundamental to cognitive development (Cook et al., 2019). EF emerges during the early years of life and continues to improve significantly through childhood, and then develops at a slower pace during adolescence (Brocki and Bohlin, 2004). Between the ages of 3–6 remains to be one of the critical times of EF development (Diamond, 2002; Carlson, 2015). Early executive functioning tends to produce a potential and delayed effect on childhood, such as academic performance and school readiness (Röthlisberger et al., 2013; Nelson et al., 2017).

The association between PA and EF is well established (Diamond and Lee, 2011; Chang et al., 2012). Although it is well known that PA facilitates cognitive development, especially EF, the degree to which PA facilitates EF substantially varies in terms of the type and quality of PA (Zach and Shalom, 2016; Shanshika et al., 2021). As for older children, adolescents, and adults, researchers suggested there is a relatively stable and positive relationship between PA and EF (Chang et al., 2012; Dan et al., 2021), while the relationship between PA and EF is inconsistent in young children, and several underlying factor may affect this relationship in preschoolers.

First, the effect of PA on cognition is related to the intensity and duration of PA. Significant dose effects have been found, which suggest longer duration and greater intensity can bring better EF improvement (Aadland et al., 2017; Egger et al., 2019; Hsieh et al., 2020; Welsch et al., 2021). However, the association between PA and EF is inconsistent in preschoolers. On the one hand, some research found that PA significantly correlated with EF development (McNeill et al., 2018; Zhang et al., 2020), while some research failed to find a positive association between MVPA and EF skills in early childhood (McNeill et al., 2018; Willoughby et al., 2018; Cook et al., 2019). Even though two studies reported that MVPA was negatively associated with performance on EF tasks (Willoughby et al., 2018; Cook et al., 2019), these results may arise from efforts to increase MVPA in early childhood which may interfere with EF skill development due to competing demands for energy expenditures related to PA and brain development (Voss et al., 2014).

The second factor is cognitive demands which are inherent in many forms of PA and induce cognitive engagement (Best, 2010; Valentin et al., 2018). Accordingly, participating in PA with cognitive demands provides individuals with opportunities of cognitive operation and training. For example, tennis playing may require adaptations for changeable conditions to improve visual-spatial skills (Gökçe and Günes, 2021); football games also stimulate attention abilities (Marianna et al., 2015). Moreover, research also indicated that motor skills contained in PA, especially complex motor skills which include multiple action changes, have positive effects in promoting individuals’ cognitive development (Valentin et al., 2016; Shafer et al., 2019). The mechanism that improves EF by cognition-engaged PA came from its complex interference environment, which places demands of executive processes as a motor action plan that must be created, monitored, and modified in the presence of continually changing tasks (Farrow and Buszard, 2017).

Another important factor lies in the different motor skill engagement in different PA, especially for young children. Motor skills, which have been defined as the ability to produce a smooth, efficient action in order to finish a particular task, have been shown to contribute to both physical health and cognitive development (Gashaj et al., 2019). Moreover, different categories of motor skills are distinguished according to reviews so far, including fine and gross motor skills, locomotor and object control skills, and body coordination (Nan et al., 2017). According to a recent research, improvements in motor competence skills are associated with improvements in EF in early childhood (Oberer et al., 2018; Willoughby et al., 2021). Tomporowski and Pesce (2019) pointed that when motor skills required for PA are complex, the individual has to process a large amount of information while controlling body movements, and therefore, EF is enhanced along with the acquisition of motor skills.

Lastly, the intervention program form should not be neglected. Interventions of group-play games seem to have better effects compared with traditional physical training. First, group play maximizes the effect of PA on EF, as most children’s PA, processed in group activities, requires complex cognition in order to cooperate with others and switching strategies to adapt to ever-changing task demands (Davis et al., 2007). Moreover, group-play intervention can also motivate children’s social involvement, and provide social learning opportunities through interaction (Eidsvg and Rosell, 2021). Cooperative and competitive exercise requires individuals to engage in multiple functions (sustained attention, working memory, regular behavior, emotional arousal such as self-esteem, stress, loneliness, etc.); the social interaction in such group play motivates individuals to do possible behaviors in response to peer reactions, and then the increased cognitive effort in this process can have beneficial effects on cognitive performance (Best, 2011; Myhre et al., 2017).

Accordingly, research (Best, 2010; Myhre et al., 2017; Zhang et al., 2020; Willoughby et al., 2021) suggested that when designing and implementing PA intervention for preschoolers, a series of factors, for example, duration, intensity, the cognition demand, and the form of PA intervention, should be taken into consideration to improve the whole effect of the PA intervention.

Therefore, in this research, a comprehensive intervention was designed and implemented for young children. The aim of this study was to determine the effects of this curriculum-prescribed MVPA intervention on EF and motor skills in 4- to 5-year-old children for 8 weeks. The group-play trial was conducted in a real-world setting where the class teachers delivered the intervention which combined MVPA with cognitive engagement in preschool children. The present research developed a group-play MVPA intervention to promote motor skills and cognition of preschoolers. In the process of intervention, the intensity and types of physical activity were controlled. In addition, we examined whether EF and motor skills between intervention and control groups differed. We hypothesized that the 8-week cognitively engaging MVPA enriching physical education would result in improvement of motor skills and core components of EF. Specifically, there are two hypotheses in this research:

H1: The group-play MVPA intervention can significantly improve the participants’ performance in preschoolers’ EF.

H2: This group-play MVPA intervention program can significantly improve the participants’ performance in preschoolers’ motor skills.

## Materials and Methods

### Participants

Participants were 62 Chinese 4- to 5-year-old preschool children (*M*_*age*_ = 4.44 years; *SD* = 0.46), who were from two classes in grade 2 of a preschool which was located in a northern city in China. To ensure the effect of regularizing the experimental response, two classes were randomly selected in the preschool, and preschoolers of one class were assigned to the intervention group (30 children, 16 boys) and preschoolers of the other class were assigned to the control group (32 children, 23 boys). Very few subjects were unable to participate in the research project consecutively due to health or family reasons, but the relevant effects were excluded by technical means during data processing.

### Design and Procedure

The group-play MVPA intervention was conducted in a real-world setting, and the measurements and intervention were carried out by the class teachers in their class. The class teachers were trained in terms of the implementation and standards of the measurements, and the teacher of the intervention group was trained with the intervention implementation procedure. All procedures performed in studies involving human participants were in accordance with the ethical standards of the institutional and/or national research committee and with the Helsinki (2000) Declaration and its later amendments; and informed consent was obtained from all individual participants included in the study.

We employed a pre-mid-post study design, and the participants of the two groups were assessed on the indexes of motor skills and dimensions of EF three times. The pretest was conducted 1 week before the intervention. The middle test was conducted in the fifth week which was in the middle of the whole intervention period. Changes in cognitive level induced by exercise persist for some time after exercise (Chang et al., 2012; Hsieh et al., 2015), therefore, this study sought to confirm whether a significant intervention effect had been achieved during the exercise intervention (4 weeks). The posttest was conducted immediately after the intervention. EF tasks (in the middle and posttests) were conducted after a break of 3–5 min by the end of the intervention. The participants of the intervention group underwent an 8-week intervention program which consisted of four cognitive-demanding MVPA games and the control group underwent the usual routine.

### Tests and Materials

#### Measurement of Preschoolers’ Executive Function

Three computer-based tasks, including the silly sound Stroop task, empty house task, and dimensional change card sorting task, were used to test the three core components of EF. The silly sound Stroop task was used to test inhibitory control. The empty house task was used to test working memory. The dimensional change card sorting task was used to measure shifting. The participants were asked to respond as quickly and accurately as possible to the computer-based procedures. The participants completed the task in the same order in a single room individually. The score of the correct reaction and the reaction time of each task were collected.

##### Inhibitory Control

The silly sound Stroop (SSS) task was used to test the children’s ability to restrain dominant responses. SSS is one of a battery of EF tasks, which retests whether the reliability and criterion validity of these tasks has been elaborated (Willoughby and Blair, 2011). The SSS task is based on a day–night task (Gerstadt et al., 1994) and it has been applied in previous research (Bull and Lee, 2014; Monette et al., 2015; Vernon-Feagans et al., 2016). A participant was required to look at two pictures (a dog and a cat) on the computer screen and click on the opposite picture according to the animal’s sound (e.g., when they heard a “meow,” they should click on the picture of a dog on the screen). Participants continuously suppressed the dominant response and clicked on the opposite picture to eliminate the interference of the dominant response. This task contained 14 practice tests with teacher’s guidance and 20 formal ones without teacher’s responses. Each point was marked when the opposite picture of every formal test was clicked on within 3 s, otherwise (wrong answers or overtime) 0 points should be marked. The score of inhibitory control ranged from 0 to 20. Researchers wrote down the subjects’ answers in a report card and the time of the formal test was recorded by a timer on a mobile phone.

##### Working Memory

The empty house task (one of the battery of EF tasks) was designed and used to test preschoolers’ working memory. The task has previously been used as an instrument to test working memory (Willoughby and Blair, 2011). In the actual implementation process, we adjusted the task according to the subjects’ performance in order to measure the visuo-spatial working memory and to reach the subjects’ existing level. The empty house task contained 5 practice tests with guidance and 15 formal tests without teacher’s responses. In each test, different numbers and positions of hippos in a Jiugong grid were shown separately on the computer screen. The preschoolers were asked to memorize the number and relative position of the hippos presented on the screen just a second ago, and then to click on the location of hippos that just disappeared on the empty Jiugong grid of the screen. As the numbers and positions of hippos changed, the memory task became more difficult. Each point was collected when all hippos of one page were found within 3 s, otherwise (wrong answers or overtime) 0 points were marked. The score of working memory formal tests ranged from 0 to 15. Researchers recorded the time of formal tests by mobile phone and wrote down both the subjects’ answers and time in a form.

##### Shifting

The dimensional change card sorting task (DCCS; Zelazo and Müller, 2007) was used to measure the shifting of the participants. There were two options (e.g., a red rabbit and a blue car) in front of a subject, and each time the subject was asked to put a card alongside one option in the same dimension. The subjects were asked to sort five cards by color, and then sort another five cards according to shape. In the third part, participants classified another 10 cards in a more difficult way (cards with/without black frame), if the card had a black frame, they should be sorted by color, if not, they should be sorted by shape. Participants received 1 point when putting one card to the right option within 3 s, otherwise (wrong answers or overtime) 0 points were marked. For example, when the teacher asked a participant to sort the blue rabbit card by color, they must put the blue rabbit card and the blue car together. The total score of DCCS ranged from 0 to 20. In the practice part, subjects had five attempts to adapt to the three different rules with teacher responses, while in the 20 formal tests, subjects had to sort cards according to three rules without guidance.

#### The Tests for Preschoolers’ Motor Skills

##### Motor Skills

Instead of the test of gross motor development (TGMD) that has been widely used as a measure of young children’s motor skills (Burns et al., 2016), we chose six tests, which including 10-m running, standing broad jump, throwing, body flexion, balance beam, and skip jump, to measure motor skills to better meet the actual situation in Chinese public kindergartens and to compare with the previous preschool studies of China. These test items are good indicators of preschoolers’ locomotor and object control skills (Wang et al., 2019; Zhang and Li, 2020; Zhang et al., 2020). All results were converted into scores ranging from 1 to 5 according to the standards in the health care work routines of Beijing preschools (5 being the best and 1 being the worst), each participant had the chance to practice twice before the tests. Therefore, the overall score ranged from 0 to 30.

### Intervention Program

The intervention lasted about 50 min each time. Training took place during work days (i.e., up to 3 days per week) throughout the intervention. In the training, participants were divided into four groups, they were encouraged to work together to win the game in the competitive conditions by gaining more scores. Meanwhile, those participants in the control group continued typical outdoor activities in the preschool as usual.

The PA intervention program was designed to promote children’s EF. In this program, the participants attended a series of physical games involving EF demands. In order to ensure exercise intensity, the researcher randomly measured the heart rate of individual children before and after each activity. The moderate to vigorous intensity heart rate should be (220-age) × 64% to (220-age) × 93%, which is 138–199 times per min (Chang et al., 2012). Before the formal exercise began, the teacher taught the children how to play. The games was played three times every week (Monday, Wednesday, Friday) during the whole 8 weeks. Participants played four games in the same exercise period, which lasted nearly 50 min each time.

In the intervention program, participants did warm up for 5 min. And then all of them played the ***‘Do the Opposite’* game** for 8 min. After taking a break for nearly 3 min, participants were divided into four groups to play ‘***Piggy Builds House’*** for 15 min. Before the third game began, they took a rest and then two groups continued to play ***‘Flip the Cards’* for 6 min.** The rest of the group (16 participants) took turns to play ‘***Mini Football game’*** for 6 min. After a short break for about 3–5 min, the four groups exchanged their positions to play the game that they had not played.

#### Do the Opposite

Children were asked to stand on the edge of a circle, while the teacher stood in the center of the circle. Children made the opposite actions according to the teacher’s commands. For example, when participants heard “girls will jump to the center,” then boys should climb to the center; when they heard “Boys, climb to the center please,” then girls should jump to the center. In this group play, participants improved skills including jumping, climbing, running, hopping, and so on. Meanwhile, they had to suppress the dominant reaction and quickly extract the corresponding actions.

#### Piggy Builds House

In this game, preschoolers were divided into four groups to compete with others to build houses by choosing building blocks of different shapes (the four groups had the same numbers and shapes of blocks). On the way to the building blocks, the children had to follow the requirements of Mother Pig (teacher), such as jumping and running back (on one foot or both feet); crawling and running back and so on. When the teacher said ‘Go,’ the first participants in the four groups competed to finish the house, followed by others in their groups who took turns to build. The winning group had the highest house by the end of the time (nearly 5 min per round).

#### Flip the Cards

Participants were divided into four groups to complete the game. Children of the same group were required to turn over the cards one by one. There were 9–12 cards in total, and one child could only turn over one card at a time (e.g., a child could only take the card and gain a point if the card was targeted, such as the number ‘8’ or ‘9,’ if not, they had to turn the card back over). The team with more points won the game. Besides, children proceeded according to the prescribed actions, which included continuous jumping with sandbags, frog jumping, and back-and-forth running.

#### Mini Football Game

Taking into account the physical conditions of children, teachers divided children into four groups (eight people in each group). And children of each group were divided into two teams to compete with each other, including three players and one goalkeeper in a team. Each round lasted about 6 min. The team with more scores won. In the football game, children coordinated and controlled their body movements in competition and cooperation, improving their skills including ball control, passing, dodging, and shooting.

### Statistical Analyses

All statistical analyses were performed using SPSS 23.0. Results were analyzed using IBM SPSS Statistics 22. Shapiro–Wilk tests for normality were preformed to justify the data and all variables did not significantly deviate from normality; accordingly, parametric tests were used in the following analyses. To examine the effect of the group-play MVPA intervention, two series of 3 (test points: pre, middle, and posttest) × 2 (conditions: intervention vs. control) repeated measures analyses of variance were conducted; through these analyses we could determine whether the two groups were equal before the intervention, moreover we could also determine whether the intervention brought different changes to the two groups.

## Results

Besides the descriptive statistics (see Table 1), two sets of analyses were conducted to examine the group-play MVPA intervention’s effect on the children’s motor skills and EF, respectively.

**TABLE 1:** Descriptive analysis of motor skills and the behavioral performance and reaction time of EF tasks.

		Pre-test		Middle test		Post-test	
	Groups	*M*	*SD*	*M*	*SD*	*M*	*SD*
**Motor skills**	Intervention group	−0.05	0.76	0.16	0.85	0.23	0.77
	Control group	0.04	0.48	−0.14	0.37	−0.20	0.35
10 meters run	Intervention group	3.33	0.92	4.23	0.90	4.33	0.71
	Control group	3.12	0.81	3.41	0.66	3.65	0.60
Standing broad jump	Intervention group	2.97	1.19	3.73	1.17	3.87	1.01
	Control group	3.62	0.92	3.82	0.72	3.91	0.67
Throwing	Intervention group	2.27	1.11	2.93	1.02	3.33	0.71
	Control group	2.62	0.78	2.91	0.57	3.17	0.46
Body flexion	Intervention group	2.20	1.16	2.77	1.14	3.06	0.87
	Control group	2.26	0.83	2.62	0.70	2.85	0.56
Balance beam	Intervention group	2.50	0.73	3.13	0.63	3.43	0.57
	Control group	2.26	0.79	2.82	0.67	3.00	0.60
Skip jump	Intervention group	2.77	1.14	3.53	1.41	3.87	1.17
	Control group	2.82	1.00	3.21	0.69	3.44	0.50
**EF (BP)**	Intervention group	0.12	0.70	0.32	0.53	0.46	0.43
	Control group	−0.11	0.86	−0.30	0.89	−0.43	0.93
Inhibitory control (BP)	Intervention group	16.87	3.38	18.47	1.93	19.43	0.90
	Control group	15.78	4.41	16.31	3.89	17.19	3.10
Working memory (BP)	Intervention group	10.43	2.57	12.37	1.81	13.47	1.41
	Control group	9.53	3.98	10.72	3.13	11.53	2.85
Shifting (BP)	Intervention group	15.47	2.56	16.67	1.90	18.10	1.58
	Control group	14.94	2.66	15.28	2.48	15.91	2.33
**EF (RT)**	Intervention group	0.03	0.85	−0.26	0.71	−0.45	0.60
	Control group	−0.03	0.72	0.24	0.79	0.42	0.76
Inhibitory control (RT)	Intervention group	72.97	12.72	63.43	9.89	55.63	7.78
	Control group	77.31	21.28	73.72	18.67	70.38	17.94
Working memory (RT)	Intervention group	71.13	26.78	56.03	17.98	45.83	13.04
	Control group	70.12	22.72	66.22	19.80	63.22	17.96
Shifting (RT)	Intervention group	99.73	29.05	79.10	16.13	69.17	14.19
	Control group	90.06	20.48	84.72	17.57	80.09	16.51

*The variable Motor is the average score of the six sub-tests scores of motor skill.*

*The variable EF BP means the average score of the z-scores of the behavioral performance on the three EF tasks.*

*The variable EF RT means the average score of the z-scores of the reaction time on the three EF tasks.*

### The Effect of the Group-Play Moderate to Vigorous Intensity Physical Activity Intervention Program on Executive Function

We used both correct behavioral performance (BP, which means the correct number) and reaction time (RT) of the participants’ reaction to the EF tasks as indexes to reflect the participants’ EF, and the two indexes were examined for the intervention effect respectively. Using BP and RT of the three EF tasks as the dependent variables, two series of 3 (test points: pre, middle, and posttest) × 2 (conditions: intervention vs. control) repeated measures analyses of variance were conducted, with the test time point as the within-subject variable, and the intervention condition as the between-subject variable (see Table 2).

**TABLE 2:** The effects of the group-play MVPA intervention program on EF and its dimensions.

	Main effects of testing point	Main effects of conditions	Interactive effect	Simple slope analysis 1	Simple slope analysis 2
Inhibitory control (BP)	*F*(2,60) = 52.64***, Δη^2^ = 0.47	*F*(1,61) = 5.14*, Δη^2^ = 0.79	*F*(2,61) = 7.71*, Δη^2^ = 0.04	C-G: *F*(2,30) = 20.39***, Δη^2^ = 0.40; T1 < T2 < T3 G-I: *F*(1,29) = 32.58***, Δη^2^ = 0.53; T1 < T2 < T3	Pretests: *t*(60) = −0.8 Mid test: *t*(60) = 2.7** Post-test: *t*(60) = 3.83***
Working memory (BP)	*F*(2,60) = 106.91***, Δη^2^ = 0.64	*F*(2,61) = 5.19*, Δη^2^ = 0.08	*F*(2,60) = 5.36*, Δη^2^ = 0.08	C-G: *F*(2,30) = 38.11***, Δη^2^ = 0.55; T1 < T2 < T3 G-I: *F*(2,30) = 68.63***, Δη^2^ = 0.70; T1 < T2 < T3	Pretests: *t*(60) = 1.05 Mid test: *t*(60) = 2.61* Post-test: *t*(60) = 3.48***
Shifting (BP)	*F*(2,60) = 88.02***, Δη^2^ = 0.60	*F*(2,60) = 5.96*, Δη^2^ = 0.09	*F*(2,60) = 18.68***, Δη^2^ = 0.24	C-G: *F*(2,30) = 23.27***, Δη^2^ = 0.43; Tl < T2 < T3 G-I: *F*(2,30) = 62.52***, Δη^2^ = 0.68; Tl < T2 < T3	Pretests: *t*(60) = 0.80 Mid test: *t*(60) = 2.46* Post-test: *t*(60) = 4.30***
Inhibitory control (RT)	*F*(2,59) = 0.09, Δη^2^ = 0.001	*F*(2,60) = 6.83*, Δη^2^ = 0.10	*F*(2,60) = 40.75***, Δη^2^ = 0.40	C-G: *F*(2,30) = 44.76***, Δη^2^ = 0.59; T1 > T2 > T3 G-I: *F*(1,29) = 92.67***, Δη^2^ = 0.76; T1 > T2 > T3	Pretests: *t*(60) = −0.97 Mid test: *t*(60) = 2.68** Post-test: *t*(60) = 4.26***
Working memory (RT)	*F*(2,59) = 0.03, Δη^2^ = 0.001	*F*(2,60) = 4.37*, Δη^2^ = 0.07	*F*(2,60) = 32.20***, Δη^2^ = 0.35	C-G: *F*(2,30) = 27.25***, Δη^2^ = 0.47; T1 > T2 > T3 G-I: *F*(1,29) = 36.36***, Δη^2^ = 0.56; T1 > T2 > T3	Pretests: *t*(60) = −0.16 Mid test: *t*(60) = 2.12* Post-test: *t*(60) = 3.34***
Shifting (RT)	*F*(2,59) = 103.59***, Δη^2^ = 0.63	*F*(2,60) = 0.24*, Δη^2^ = 0.004	*F*(2,60) = 28.10***, Δη^2^ = 0.32	C-G: *F*(2,30) = 34.79***, Δη^2^ = 0.53; T1 > T2 > T3 G-I: *F*(1,29) = 68.85***, Δη^2^ = 0.70; T1 > T2 > T3	Pretests: *t*(60) = −0.80 Mid test: *t*(60) = −2.46* Post-test: *t*(60) = −4.30***

*p < 0.05, **p < 0.01, ***p < 0.001.

*BP, behavioral performance; RT, reaction time.*

First, regarding the participants’ BP of the three EF components, all the main effects of testing points and conditions were significant; in addition, significant time points by condition interactions were also observed for all the BP indexes. When the main effect and interaction effect are both significant, only the interactive effect should be analyzed with simple slope analyses. We analyzed these interactive effects from two aspects. First, we split the sample into the control group and intervention group, and conducted repeated measures analyses of variance to find out whether EF components differed between the three time points. The result indicated that, for both groups, there were significant differences between the three time points, with the scores of the posttests significantly higher than those of the middle tests, and the scores of the middle tests significantly higher than those of the pretests. Second, by conducting a series of independent *t*-tests, we compared the scores of EF components in pre, middle, and posttests to explore the difference between the intervention and control groups. The results suggested that, for all the three BP indexes, the intervention and control groups were equal at the pretest, but significantly differed at the mid and posttests. In sum, although EF dimensions of both the control and intervention groups increased during the 8 weeks, the intervention group presented a more rapid and greater growth than the control group.

Similar analyses were conducted to the RTs of the three EF tasks. As Table 2 suggests, all the three time points by condition interactive effects were significant, and the following simple-slope analyses were examined from two aspects. First, the results suggested that repeated measures analyses of variance were conducted, and the results showed that RTs decreased significantly with time points in both the control and intervention groups. Second, independent *t*-tests were conducted to compare the scores of EF tasks between the control and intervention groups in pre-, mid, and post-tests, and the results suggested that, for all the three EF components, there was no significant difference between the two groups, a significant difference emerged at the middle test, and the difference between the intervention group and control group were very significant in the posttests. That is to say, the index of RT also showed similar results: although RTs of the intervention group and control group showed a significant trend of decrease, the intervention group decreased more rapidly.

In brief, using indexes of BP and RT, the above analyses suggested that, although EF components developed significantly in both the intervention group and control group, the intervention group had a higher increasing amount for both the indexes. These results proved that the group-play MVPA intervention is effective in facilitating preschoolers’ EF.

### The Effect of the Group-Play Moderate to Vigorous Intensity Physical Activity Intervention on Motor Skills

We also examined the effect of the group-play MVPA intervention on motor skills by a series of repeated measures analyses of variance. The results are summarized in Table 3.

**TABLE 3:** The effects of the group-play MVPA intervention program on motor skills and its dimensions.

	Main effects of testing point	Main effects of conditions	Interactive effect	Simple slope analysis 1	Simple slope analysis 2
Motor skills	*F*(2,63) = 184.78**, Δη^2^ = 0.746	*F*(1,61) = 0.08, Δη^2^ = 0.001	*F*(2,61) = 14.08**, Δη^2^ = 0.183	G1: *F*(1,33) = 81.09***, Δη^2^ = 0.71 T1 < T2 < T3 G2: *F*(1,29) = 102.14***, Δη^2^ = 0.77 T1 < T2 < T3	Pretest: *t*(63) = 1.17 Mid test: *t*(63) = −0.78 Post-test: *t*(63) = −1.25
10-meter running	*F*(2,63) = 62.76**, Δη^2^ = 0.503	*F*(1,61) = 0.08, Δη^2^ = 0.149	*F*(2,61) = 9.82**, Δη^2^ = 0.137	G1: *F*(1,33) = 15.98***, Δη^2^ = 0.33 T1 < T2 < T3 G2: *F*(1,29) = 50.43***, Δη^2^ = 0.64 T1 < T2 < T3	Pretest: *t*(62) = −1.00 Mid test: *t*(62) = −4.21** Post-test: *t*(63) = −4.20***
Standing broad jump	*F*(2,63) = 40.62**, Δη^2^ = 0.396	*F*(1,61) = 1.35, Δη^2^ = 0.021	*F*(2,61) = 1.07 Δη^2^ = 0.156	N/A	Pretest: *t*(62) = 1.46 Mid test: *t*(62) = 0.38 Post-test: *t*(63) = 0.21
throwing	*F*(2,63) = 53.66**, Δη^2^ = 0.46	*F*(1,61) = 0.105, Δη^2^ = 0.002	*F*(2,61) = 5.56**, Δη^2^ = 0.082	G1: *F*(1,33) = 18.75**, Δη^2^ = 0.36 Tl < T2 = T3 G2: *F*(1,29) = 33.11***, Δη^2^ = 0.53 Tl < T2 = T3	Pretest: *t*(62) = 1.48 Mid test: *t*(62) = −0.11 Post-test: *t*(63) = −1.11*
Body flexion	*F*(2,63) = 52.41**, Δη^2^ = 0.458	*F*(1,61) = 0.23, Δη^2^ = 0.004	*F*(2,61) = 1.06, Δη^2^ = 0.032	N/A	Pretest: *t*(62) = 0.26 Mid test: *t*(62) = 0.64 Post-test: *t*(63) = −1.19
Balance beam	*F*(2,63) = 74.14**, Δη^2^ = 0.545	*F*(1,61) = 4.91*, Δη^2^ = 0.073	*F*(2,61) = 2.00*, Δη^2^ = 0.03	G1: *F*(1,33) = 35.50***, Δη^2^ = 0.52 Tl < T2 < T3 G2: *F*(1,29) = 38.07***, Δη^2^ = 0.57 Tl < T2 < T3	Pretest: *t*(62) = −1.23 Mid test: *t*(62) = −1.90^†^ Post-test: *t*(63) = −2.95**
Skip jump	*F*(2,63) = 46.84**, Δη^2^ = 0.430	*F*(1,61) = 1.00, Δη^2^ = 0.016	*F*(2,61) = 4.00**, Δη^2^ = 0.06	G1: *F*(1,33) = 19.14***, Δη^2^ = 0.37 Tl < T2 < T3 G2: *F*(1,29) = 26.47***, Δη^2^ = 0.48 Tl < T2 < T3	Pre-test: *t*(62) = 0.21 Mid test: *t*(62) = −1.21 Post-test: *t*(63) = −1.93^†^

*p < 0.05, **p < 0.01, ***p < 0.001. 0.05 <†< 0.1

As Table 3 indicates a set of 3 (test points: pre, middle, and posttest) × 2 (conditions: intervention vs. control) repeated measures analyses of variance were conducted which made the six motor skill indexes (10-m running, standing broad jump, throwing, body flexion, balance beam, and skip jump) dependent variables. The test point was the within-subject variable and the condition was the between-subject variable. Except the standing broad jump and body flexion, the other four motor skill indexes had a significant interaction between the two variables. We also used two types of simple slope analyses to examine these interactive effects. In the first simple slope analysis, we split the sample into the intervention group and the control group, and examined the scores of the pre, middle, and posttests with repeated variance analyses in each group. It was found that for the six components of motor skills, the scores of both groups increased significantly with the test points. In the second simple slope analysis, we compared the scores of the two groups in each of the three time points, and found that, for the scores of throwing and skip jump, the intervention and control groups were equal at the pre and middle tests, however, the intervention group scored higher than the control group in the posttests. For 10-m running and balance beam, the two groups were equal at pretest, but the intervention group scored higher than the control group in the middle and posttests. Taken together, although the control group showed a significant developing trend in the six components of motor skills, the intervention group exhibited a greater improvement.

In summary, the above analyses proved the special effectiveness of the group-play MVPA intervention on both the development of motor skills and EF components.

## Discussion

The present research examined the effect of a group-play MVPA intervention program on the development of motor skills and EF in 4- to 5-year-old preschoolers. Overall, 62 preschoolers of 4–5 years old participated in the pilot cluster randomized controlled trial to examine the effectiveness of this program, and the result indicated that both the preschoolers’ motor skills and EF improved significantly.

To begin with, this group-play MVPA intervention improved the preschoolers’ motor skills significantly, and this result was in accordance with previous research where PA improved individuals’ motor skills and capability (Zeng et al., 2017; Sansi et al., 2021), including preschoolers, because PA provided individuals with the opportunity to exercise their motor skills (Figueroa and An, 2017). Another study indicated that PA with the special purpose of practicing certain movements brought significantly more benefits to young children’s motor skill development (Coutinho et al., 2016). And vice versa, the improvement of motor skills also increased children’s tendency to participate in PA (Dapp et al., 2021). Recent research further proved that compared with low intensity PA, MVPA had an additional positive effect on preschoolers’ motor skills (Nilsen et al., 2020).

Moreover, the relationship between PA and cognition development in young children has been well examined. However, how to develop an appropriate intervention program is still a challenge to both researchers and practitioners all over the world. In this research, we tried to integrate three characteristics of PA, which have been found to play a critical role in young children’s cognition development, into a preschool-based PA intervention. These characteristics were the intensity and duration of PA (Egger et al., 2019), cognitive load (Aadland et al., 2017; Willoughby et al., 2021), and the form of the games (Myhre et al., 2017; Eidsvg and Rosell, 2021); and by integrating these factors, we designed an 8-week group-play MVPA intervention for cognition in children. Our hypothesis that the program is effective in promoting preschoolers’ EF has been supported. How this intervention facilitated the development of motor skills and EF in preschoolers is discussed in the following.

First, the effectiveness of the intervention program may partly stem from the moderate to vigorous intensity physical activity. It is well established that MVPA has positive effects on cognitive development in mostly older children (Egger et al., 2019), adolescents (Koorts et al., 2019), and adults (O’Brien et al., 2021). However, some research studies also found converse results where MVPA decreased the preschoolers’ performance on EF tasks (McNeill et al., 2018; Willoughby et al., 2018; Cook et al., 2019); the reason may be that intense PA consumes cognitive processing resources, which are especially limited in young children; therefore, MVPA will decrease children’s cognitive performance (Voss et al., 2014). The present intervention program confirmed that the MVPA training contributed to the increase of EF, and it may result from two things. The first reason may be that, instead of continuously, the preschoolers in this program participated in MVPA intermittently and consumed relatively fewer cognitive resources. The second reason may be that, as previous research indicated that positive emotion had a positive effect on individuals’ EF (Wang et al., 2017), the familiar, natural, and cheerful context in the present program may make children feel relaxed and happy, and consequently contribute to the preschoolers’ improved performance of EF tasks.

Second, another explanation to the effectiveness may be that the present intervention improved EF of preschoolers with the involvement of cognitive operation. Plenty of research studies have proved that the cognitive load in PA is positively related to executive development (Miller and Best, 2010; Valentin et al., 2018). In this intervention program, we increased the cognitive load in the intervention tasks, and almost all tasks required children to use components of EF. For example, in the “Flip the Cards” game, participants had to use visuo-spatial working memory to remember the card position; and during the process, the participants practiced and improved their visuo-spatial working memory. Another case is “Do the Opposite,” in order to help preschoolers to develop inhibitory control, the participants were required to overcome their dominant reactions and to do the opposite actions to the teachers’ orders. Moreover, reams of research indicated that complicated motor movement also added to the cognitive load of PA (Aadland et al., 2017; Tomporowski and Pesce, 2019; Willoughby et al., 2021). In our intervention program, the children had to complete a lot of complicated gross (combination of walk, run, jump, throw, and kick) and fine movements (pinch, hold, point with one’s fingers); these movements may also facilitate children’s cognitive engagement in the intervention program and consequently improve their cognitive development.

Third, this intervention program was implemented through group-play games, which might also contribute to EF development in the preschoolers. In group play, children had to cooperate and compete with their peers, which may produce an exceeding effect of improving EF beyond the single exercise. The function of group play may be that the social interaction required the preschooler to respond to their peers’ behaviors on time and predict their following actions, which will demand more cognitive efforts (Best, 2011; Myhre et al., 2017). In each of the four games, the children had to interact with peers closely; for example, in the ‘Piggy Builds House’ game, the children needed to determine how to choose appropriate blocks according to their peers’ building situation.

## Limitations and Future Directions

Due to the constraints of the study setting, the measurement tools chosen for the study for EF and motor skills have yet to be refined. Accordingly (Miyake and Friedman, 2012), the task impurity of the instrument is a problem, any score derived from an EF task necessarily includes systematic variance attributable to non-EF processes (e.g., articulation speed). It can be alleviated by using multiple measures of each EF component under investigation (Snyder et al., 2015).

This study did not use various tasks to investigate different EF components. Panesi et al. (2022) argued that tests containing an updating task for preschoolers should also be included due to the distinction between working memory capacity and working memory updating. Moreover, a test for inhibitory control requires a novel response in the face of a conflicting prepotent response (conflict scale) (e.g., children have to overcome dominant responses in the SSS task to select the opposite image) and the delay of a prepotent response (delay scale) (e.g., the gift delayed task, Kochanska et al., 2000; Carlson and Moses, 2010). To greatly avoid the impact of task impurity on the results, the study should also make adjustments in the choice of EF.

Instead of the test of gross motor development (TGMD) that has been widely used as a measure of young children’s motor skills (Burns et al., 2016), we used other motor tests, including 10-m running, standing broad jump, throwing, body flexion, balance beam, and skip jump, to measure motor skills. The six tests were originally chosen to facilitate comparison with similar studies in China, but we are fully aware of their limitations and that more scientific and specialized testing tools should be used.

As for subjects, aiming to ensure the scientific validity of the data, the study should test cognition, neurodevelopmental disorders, and sensory functions to control for effects of extraneous variables on testing. Moreover, this study should fully consider the issue of individual differences in motor skills and cognition within the same group, and divide the preschoolers, according to the results of motor skills and EF, into different groups before comparing intervention and control groups. Besides, considering the age and gender differences in EF and motor skills of preschool children is also of importance (Carlson and Moses, 2010; Jacobsen et al., 2017); due to practical issues, age of children and the number of boys in the intervention and control groups differed, the age and number of boys and girls in the subjects of the two groups should be balanced.

On the other side, this study did not include two control groups. If the situation was ideal, two control groups should be selected. On the one hand, it could better eliminate the impact of different teaching styles of teachers. On the other hand, it may include a control group of the lowest level of cognitive engagement of MVPA or high-level cognitive PA with moderate intensity, so as to compare the different effects on EF between various levels of cognitively engaged MVPA or between the same cognitive demand with different intensities of PA.

In addition, feelings of participants from the intervention group and control group should also be tested immediately after each session, so as to better verify the relationship among intervention, EF, and children’s mood. It is a good way to study the effect of emotion which can serve as an intermediate variable on motor skills and EF. Finally, the study should also exclude the influence of family conditions, including the influence of family income, parent education level, second-child family, and so on. Moreover, it is worthwhile to test the consistency and temporary effects of being cognitively engaged in MVPA on cognition. In the present study, the frequency and time points of EF tests were not covered over the whole intervention period, such as testing EF after each session and a delay of 10–20 min or testing in the day without a session, or even 5 days after the whole intervention was completed.

## Conclusion

Regarding the positive effects on preschooler’s EF and motor skills, one might ask how significant this finding is for the educational setting in general and what possible consequences it may have for PA at preschool. The intervention of this study can be used in preschool physical education to improve motor skills and cognitive functions of young children. This study provides a new perspective for preschool teachers to design and organize outdoor sports activities, and help teachers focus more on the cognitive benefits of cognitively challenging PA for preschoolers. In addition to refining teachers’ theoretical conceptions, the study also provides implications for teachers when making plans on cognitive engagement PA including increasing the frequency of movement changes and possibility of cooperative and competitive group play in PA and adding tasks related to EF in sports activities to improve the promoting effect of physical activities on cognition. Further research should focus on studying the relationships between different intensities of PA with low-high cognitively engaged group play interventions, as well as their internal mechanisms.

## Data Availability Statement

The original contributions presented in this study are included in the article/supplementary material, further inquiries can be directed to the corresponding author.

## Ethics Statement

The studies involving human participants were reviewed and approved by the Ethics Committee in Capital Normal University. Written informed consent to participate in this study was provided by the participants’ legal guardian/next of kin.

## Author Contributions

JB was involved in experimental method design, actual investigation and research, experimental data analysis, visualization of experimental results, and writing with the first draft of the manuscript. HH was mainly involved in research concept generation, research funding acquisition, research resource collection, experimental design validation and verification, research project supervision and guidance, and manuscript review and revision. HO was mainly responsible for the polishing and expression of words and manuscript review and revision. All authors contributed to the article and approved the submitted version.

## Conflict of Interest

The authors declare that the research was conducted in the absence of any commercial or financial relationships that could be construed as a potential conflict of interest.

## Publisher’s Note

All claims expressed in this article are solely those of the authors and do not necessarily represent those of their affiliated organizations, or those of the publisher, the editors and the reviewers. Any product that may be evaluated in this article, or claim that may be made by its manufacturer, is not guaranteed or endorsed by the publisher.
